# Endocardial focal activation originating from Purkinje fibers plays a role in the maintenance of long duration ventricular fibrillation

**DOI:** 10.3325/cmj.2014.55.121

**Published:** 2014-04

**Authors:** Changjian Lin, Qi Jin, Ning Zhang, Jian Zhou, Yang Pang, Yangxun Xin, Shaohua Liu, Qiong Wu, Liqun Wu

**Affiliations:** Department of Cardiology, Shanghai Rui Jin Hospital, Shanghai Jiao Tong University School of Medicine, Shanghai, P.R. China

## Abstract

**Aim:**

To determine the role of repetitive endocardial focal activations and Purkinje fibers in the maintenance of long duration ventricular fibrillation (LDVF, VF>1 minute) in canine hearts in vivo.

**Methods:**

The study was conducted in electrophysiological laboratory of Shanghai Ruijin hospital from July 2010 to August 2012. A 64-electrode basket was introduced through a carotid artery into the left ventricle (LV) of 11 beagle dogs for global endocardial electrical mapping. In the Lugol’s solution group (n = 5), the subendocardium was ablated by washing with Lugol’s solution. In the control group, (n = 6) saline was used for ablation. Before and after saline or Lugol ablation, we determined QRS duration and QT/QTc interval in sinus rhythm (SR). We also measured the activation rates in the first 2 seconds of each minute during 7 minutes of VF for each group. If VF terminated spontaneously in less than 7 minutes, the VF segments used in activation rate analysis were reduced accordingly.

**Results:**

At the beginning of VF there was no difference between the groups in the activation rate. However, after 1 minute of LDVF the Lugol’s solution group had significantly slower activation rate than the control group. In the control group, all episodes of LDVF (6/6) were successfully sustained for 7 minutes, while in the Lugol’s solution group 4/5 episodes of LDVF spontaneously terminated before 7 minutes (4.8 ± 1.4 minutes) (*P* = 0.015). In the control group, at 5.1 ± 1.3 minutes of LDVF, a successive, highly organized focal LV endocardial activation pattern was observed. During this period, activations partly arose in PF and spread to the working ventricular myocardium. Mapping analysis showed that these events were consistent with repetitive endocardial focal activations. No evidence of similar focal activations was observed in the Lugol’s solution group.

**Conclusions:**

Repetitive endocardial focal activations in the LV endocardium may be associated with activation of subendocardial PFs. This mechanism may play an important role in the maintenance of LDVF.

Sudden cardiac death (SCD), commonly caused by ventricular fibrillation (VF), is responsible in China for at least 500 000 deaths per year (1). Typically, patients with SCD due to VF are not defibrillated for several minutes, even in areas with the shortest first response times. After 10 minutes of VF, few patients are successfully resuscitated (2). However, after 5 minutes of VF, cardiopulmonary resuscitation (CPR) for 3 minutes prior to the first shock resulted in 22% survival to hospital discharge and 4% survival when the defibrillation shock was given before CPR (3). Thus, survival after more than 5 minutes of VF is possible. Furthermore, different treatment algorithms may be preferable during different durations of long duration ventricular fibrillation (LDVF). As such, it is important to understand the mechanisms of LDVF maintenance. However, despite our understanding of VF initiation and early VF, the electrophysiological mechanism by which LDVF is maintained remains largely unknown (4).

Early VF is characterized by rapid, chaotic activation, after which a more organized pattern is observed (5,6). Although the primary VF maintenance mechanism regardless of VF duration is considered to be reentry into the working ventricular myocardium (WVM) (4,7), the mechanisms for short duration VF (SDVF, VF<1 minute) and LDVF may differ (4,7,8). While in SDVF the dominant driving force is reentrant activity, in LDVF it may be Purkinje fiber (PF) activation (4,7,8). For example, Tabereaux et al (9) reported highly active PFs throughout the first 10 minutes of VF using an isolated canine heart model of repetitive endocardial focal activations during LDVF. Furthermore, these PF were associated with the genesis of repetitive endocardial focal activations. Thus, the aim of the present study was to test whether repetitive endocardial focal activations played a role in the maintenance of LDVF, and to determine the role of PF in LDVF maintenance in canine hearts in vivo.

## Materials and methods

This study was conducted from July 2010 to August 2012. Eleven one-year old male beagles (10-12 kg) were purchased from Shanghai Jiao Tong University Agriculture College and raised under controlled conditions at the Department of Animals for Scientific Research, Shanghai Jiao Tong University School of Medicine. All procedures were approved by the Animal Protection Committee of Shanghai Jiao Tong University.

### Animal preparation

The animals were injected intramuscularly with ketamine (10 mg/kg) and atropine (0.04 mg/kg) for anesthetic induction. Anesthesia was maintained with propofol (8-16 mL/kg/h), and animals were restrained and ventilated in a dorsally recumbent position. Arterial blood gasses, arterial blood pressure, electrocardiogram, and serum electrolytes were monitored and maintained within the reference ranges throughout the study. The heart was exposed through a median sternotomy and supported in a pericardial sling. A 31-mm multielectrode basket (Constellation, Boston Scientific EP Technologies, Watertown, MA, USA) was introduced through a carotid artery into the left ventricle (LV) for global endocardium electrical mapping. A catheter (model 80993; IBI, St. Jude Medical, Saint Paul, MN, USA) with one electrode in the right ventricle and the other electrode in the superior vena cava was inserted for defibrillation.

### Subendocardium ablation procedure

In the control group, the LV subendocardium was flushed with saline (n = 6) and in the experimental group with Lugol's solution (5 g I_2_ and 10 g KI dissolved in 100 mL deionized H_2_O; n = 5). One rubber thong was twisted around the pulmonary arteries and veins to prevent the communication between the pulmonary circulation and the LV. Another rubber thong was twisted around the aortic artery to occlude the LV outflow tract. Through the left femoral artery access, a catheter was retrogradely positioned through the aortic valve into the LV apex by fluoroscopic guidance for drawing blood and solution injection. VF was induced by 30 Hz current (MicroPace III, EPS 320 Cardiac stimulator, Micropace EP Inc., Santa Ana, CA, USA) delivered through the pacing electrode in the mapping basket.

After the blood in the LV cavity was fully drawn out using a 50 mL syringe, the LV cavity was flushed with saline or Lugol's solution. After 10 seconds, saline or Lugol's solution was withdrawn and the ventricular cavity was fully irrigated with warm normal saline through a catheter. The rubber thongs were then released. A biphasic (6/4 ms) shock (30 J) was delivered from a defibrillator (Teletronic Pacing Systems, 4510 Implant Support Device, Teletronics Pty Limited, Malvern, Australia) via catheter electrode to terminate VF. The total ischemic time was kept to less than 2 minutes. The dogs were allowed to recover for at least 15 minutes, until blood pressure and heart rate returned to steady state. QRS duration and QT/QTc interval were determined in sinus rhythm (SR) before and after saline or Lugol ablation.

### Signal analysis

PF and WVM activations were identified in the 64-electrode basket recordings using software and manually rechecked. PF activations were identified and distinguished from WVM activations with an algorithm similar to that previously described (10). The two-point dV/dt was calculated as follows: [V(n +1) − V(n)]/t. WVM activations were determined when dV/dt was the most negative during the complexes that were >4 ms long and when dV/dt was more negative than -0.5 mV/ms. PF activations were identified when dV/dt was the most negative during the complexes that were ≤4 ms long and when dV/dt was more negative than -0.1 mV/ms.

### Long-duration ventricular fibrillation analysis

A 7-minute LDVF was induced by 30 Hz current delivered through the pacing electrode in the mapping basket. The electric signals were recorded with a 160 channel, 2 kHz sampling rate mapping system, and unipolar electrograms were amplified and band pass-filtered between 0.5 Hz and 1 kHz. Activation rates were measured in the first 2 seconds of each minute. If VF terminated spontaneously in less than 7 minutes, the VF segments used in activation rate analysis were reduced accordingly. When VF continued for more than 7 minutes, internal defibrillation was used to stop it. Ventricular pacing and re-induction of VF following VF termination were performed to evaluate the excitability of the ventricles.

Repetitive activity was defined as an activation pattern that was repeated for ≥2 consecutive beats (11). We used repetitive endocardial focal activations to describe the repetitive activities observed at the LV endocardium. At the end of the experiments, all animals were sacrificed and their hearts were fixed and routinely processed. Transmural sections of the LV tissues for each heart were obtained and stained with hematoxylin and eosin to determine the extent of myocardial injury caused by Lugol’s solution.

### Statistical analysis

Statistical analysis was conducted using SPSS 16.0 for Windows (SPSS Inc., Chicago, IL, USA). Data are expressed as mean ± standard deviation. Normality of distribution was tested by Shapiro-Wilk test. Paired *t* tests were performed to compare the QRS duration and QT/QTc interval before and after Lugol ablation. Differences in activation rate between control and Lugol’s solution group were determined by independent-samples *t* tests. Due to the small sample size, Fisher exact test was used to access the ability of LDVF sustaining for 7 minutes. *P* < 0.05 was considered significant.

## Results

### Basic electrophysiological parameters

**Figure 1:**
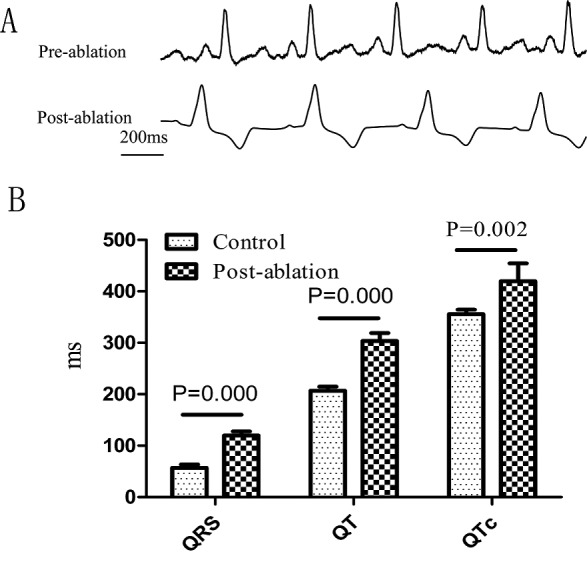
Basic electrophysiological parameters pre-ablation and post-ablation. (**A**) Actual recordings of lead II of the surface ECG. After Lugol ablation, a morphology indicative of left bundle branch block pattern immediately developed and (**B**) QRS duration and QT/QTc interval were significantly prolonged.

There was no significant difference in age between the two groups. In the Lugol’s solution group, all animals regained sinus rhythm after ablation. Compared with the baseline, the body surface electrocardiogram immediately indicated left bundle branch block (Figure 1A). Lugol ablation significantly increased the QRS duration by 110% (*P* < 0.001) during SR and significantly lengthened the QT and QTc interval compared with the baseline (47% and 56%, respectively; *P* < 0.001 and *P* = 0.002) (Figure 1B).

### Endocardial recordings

**Figure 2:**
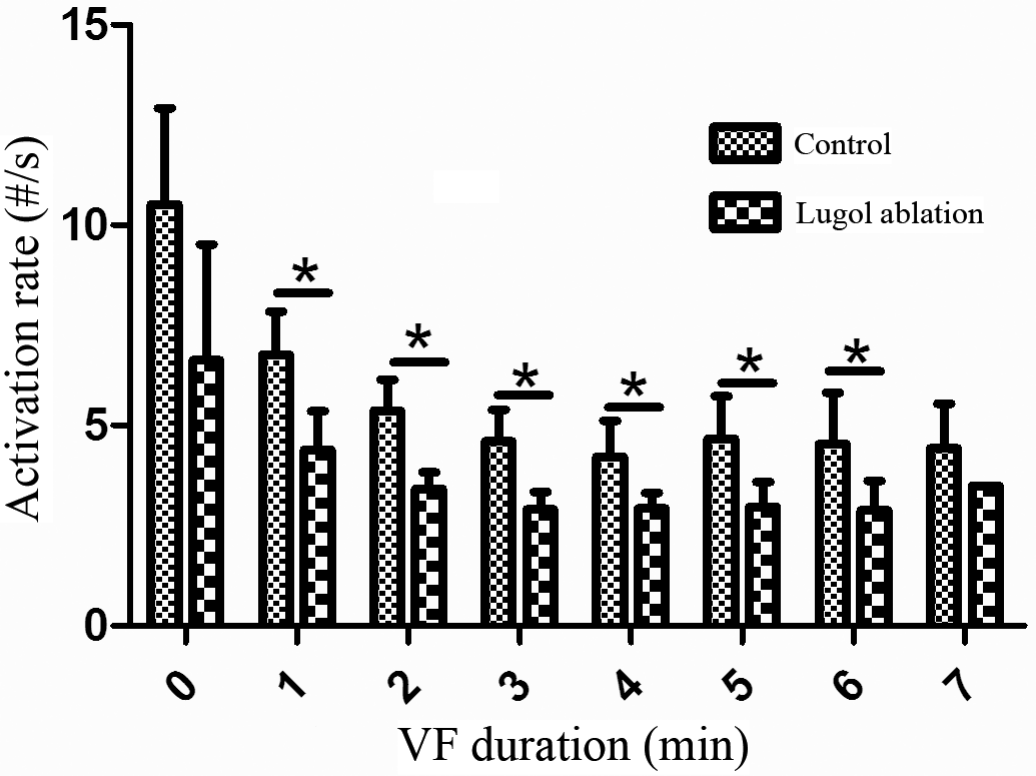
Activation rates of the 64-electrode basket. Average activation rates (mean with bars showing standard deviation) for Lugol's solution and control group. Significant differences are denoted with asterisks. The mean activation rates of 1-6 minutes of ventricular fibrillation (VF) were significantly slower in the Lugol's solution than in the control group. Statistical analysis was not performed for the 7 minutes data as VF persisted in only one heart in the Lugol's solution group in this period.

**Figure 3:**
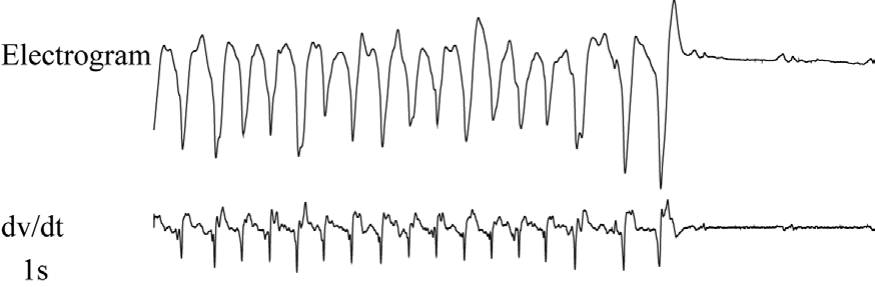
Electrical activations were recorded until long duration ventricular fibrillation (LDVF) abruptly terminated. Electrogram (top) and its temporal derivative (bottom) from a basket in a Lugol-ablated heart demonstrate the spontaneous termination of LDVF. No Purkinje activations were detected.

PF and WVM activations were recorded in control group animals during SR and LDVF. After Lugol ablation, PF activations were no longer observed. At the beginning of VF, there was no significant difference in the activation rate between the groups (*P* = 0.052). However, after 1 minute of LDVF, the Lugol’s solution group had significantly slower activation rate than the control group (1 min, *P* = 0.006; 2 min, *P* = 0.003; 3 min, *P* = 0.005; 4 min, *P* = 0.031; 5 min, *P* = 0.021; 6 min, *P* = 0.030) (Figure 2). In the control group, all LDVF episodes (6/6) were successfully sustained for 7 minutes, while in the Lugol’s solution group 4/5 episodes spontaneously terminated before 7 minutes (4.8 ± 1.4 minutes) (*P* = 0.015) (Figure 3). Sinus rhythm was regained in all animals in the control group and in 3/5 animals in the ablation group. After the termination of LDVF, the ventricles of both groups were excitable.

### Repetitive endocardial focal activations

**Figure 4:**
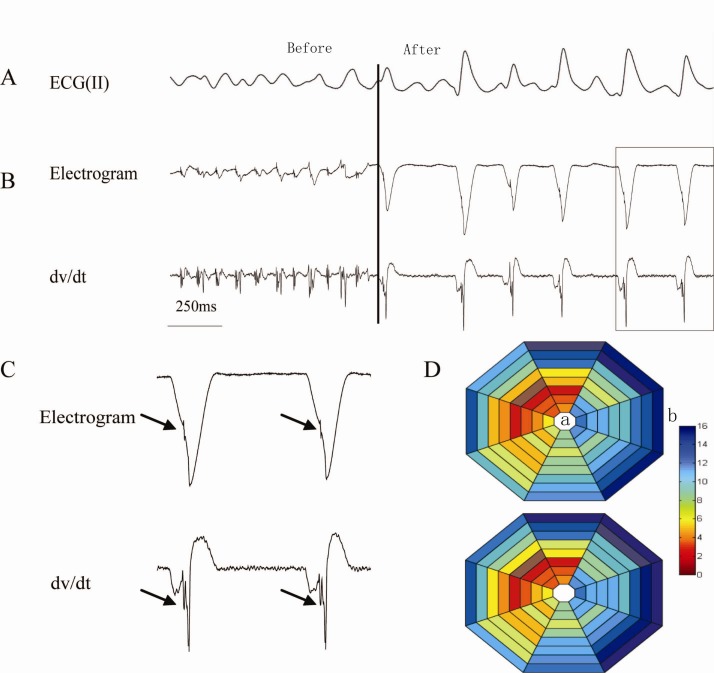
Examples of repetitive endocardial focal activations during long duration ventricular fibrillation (LDVF). (**A**) ECG continued to exhibit a polymorphic ventricular tachycardia-like pattern (left to the vertical line), while an organized activation pattern was present on the endocardium (right to the vertical line). (**B**) Electrogram (top) and its temporal derivative (bottom) from a basket in a control group heart. LDVF changed abruptly from a disorganized activation pattern to an organized activation pattern. The box in B represents the selected beats displayed in Figure 4. (**C**) Beats in more detail. Arrows indicate PF activations. (**D**) 64-electrode basket orientation in the LV. The catheter contained eight splines each with eight electrodes (triangles). Apical electrodes are located toward the center of the display (**a**) and basal electrodes toward the periphery (**b**). Activation times are indicated by the color bar. The electrical mapping was consistent with repetitive endocardial focal activations, and the earliest activation was in the septal-basal electrodes.

In the control group, a successive, highly organized focal LV endocardial activation pattern was observed at 5.1 ± 1.3 minutes of LDVF (Figure 4B). During this period, activations partly arose in the PF and spread to the WVM. Mapping analysis showed that these events were consistent with repetitive endocardial focal activations (Figure 4D). There was no evidence of repetitive endocardial focal activations in the Lugol’s solution group. The earliest PF activation occurred in the septal-basal electrodes in the control group . In the Lugol’s solution group, there was no evidence of endocardial focal activation originating from PF.

### Histological findings

In the Lugol’s solution group, 5 serial transmural sections were examined. Myocardial fibers showed both stretching and waviness, and the subendocardium showed acute necrotic changes including cytoplasmic homogeneity and pyknosis. These histological findings were similar to those reported in previous canine studies (12-14).

## Discussion

The major findings of the current study were that the Purkinje system ablation caused a prolonged QRS duration and QT/QTc interval, a significant decrease in the VF activation rate after 1 minute of VF, earlier spontaneous termination of LDVF, and disappearance of the repetitive endocardial focal activations in the control group during LDVF. These data suggest that repetitive endocardial focal activations at the LV endocardium might be associated with activation from the subendocardial PF, which in turn may play an important role in the LDVF maintenance.

PF are specialized to rapidly conduct impulses. Ventricular discharge through the Purkinje system is defined as a standard electrocardiogram QRS complex, which corresponds to depolarization of the right and left ventricles. The QT interval represents electrical depolarization and repolarization of both ventricles. Electrical impulse propagation was reported to occur significantly faster in PF than in WVM (0.3-0.4 mm/ms vs 2-3 mm/ms) (15). In the present study, PF in the LV were destroyed by Lugol ablation of the subendocardium, which led to propagation of the activation wave front from the WVM to the WVM, but not from the PF to the WVM. Therefore, the time required for LV depolarization was prolonged. Accordingly, the QRS duration after ablation was significantly longer than at the baseline. Also, a signal morphology indicative of left bundle branch block immediately developed in the body of ECG.

The VF activation rate in both groups slowed down gradually and decreased significantly after 1 minute of VF following Purkinje system ablation. These data suggest that although reentrant activity in the WVM might be the dominant driver of activation during the first minute of VF (16), PF activation plays an important role in wave front activation after 1 minute of LDVF.

In LDVF the Purkinje system is activated more rapidly than WVM, as PF are more resistant to global ischemia during VF than working myocardial cells (17-20). This may be due to the increased metabolic load required for the contractions of myocardial cells and to the increased glycogen stored in PF. Furthermore, in dogs as well as in humans, the Purkinje system is situated near the endocardium and thus receives a lesser insult than the WVM because of oxygen diffusion from the nearby LV cavity. In addition, although the refractory period of PF in SR is longer than for myocardial cells, it is shorter when PF is activated rapidly. The rapid activation rate observed in VF may reduce the refractory period of PF below that for the WVM, thus enabling PF to activate faster (21). Therefore, after Lugol ablation, when the Purkinje activations were entirely eliminated within the mapped regions, a change in the LDVF activation rate was observed.

In the Lugol’s solution group, activation rates in four animals slowed down to approximately 2/s and then spontaneously terminated, while in no control animal activation rates slowed down to 2/s or terminated during 7 minutes of LDVF. There may be a minimum required activation rate for sustaining the intramural reentry, which is considered important in maintaining VF (22-24). In pigs, the percentage of wave fronts that appeared de novo in the WVM increased with increasing duration of VF. Since in these animals, the Purkinje-muscle junctions are extended from the endocardium almost to the epicardium, the Purkinje system is a likely source of activation fronts (8). As such, the Purkinje system may play a crucial role in sustaining LDVF by maintaining a minimum activation rate required for intramural reentry to maintain VF.

Tabereaux et al reported in an isolated canine heart model with a relatively small mapped area that PF were highly active throughout the first 10 minutes of VF (9). Using the same model, Dosdall et al ablated the Purkinje system with Lugol’s solution and found that LDVF was spontaneously terminated earlier than in the control group (25). However, this perfused isolated heart model did not include autonomic system control, which is known to alter outcomes in intact hearts and clinical VF (9). In the present study, we employed an in vivo heart model and found that the Purkinje system played an important role in the maintenance of LDVF.

A number of studies have described different phases of activity in the first several minutes of VF (5,11,26,27). Early VF is characterized by rapid, chaotic activation, followed by a brief period of greater organization, with larger and more repetitive wave fronts. Panoramic optical mapping of continuously perfused, non-ischemic, fibrillating swine hearts demonstrated that most wave fronts were continuous and persistent throughout the mapped episodes (28); non-epicardial sources of activation were not necessary for VF maintenance. Although this may be true for SDVF or perfused VF, the incidence of epicardial reentry decreases with progression of global ischemia (5). After several minutes of unperfused VF, there is a period of increased conduction block, smaller wave fronts, and slower activation rates (5,11). Although early VF is driven primarily by reentrant activity in the WVM, these changes in VF characteristics suggest that VF persisting for more than a few minutes may be driven by an alternative mechanism.

Similarly to Tabereaux et al (9), we found a frequent focal activation during LDVF that arose in either PF or WVM. They further found that 42% of the focal wave fronts arose in the PFs, and in 82% of these wave fronts, the PF activation appeared to propagate into and initiate a WVM activation. Using electrical mapping, we also observed the repetitive endocardial focal activation phenomenon that was described previously in isolated rabbit hearts during LDVF. This study found that the repetitive endocardial focal activations appeared as two activation patterns (ie, with or without a “narrow-wave” pattern) (29). Although the underlying mechanism of repetitive endocardial focal activations with a “narrow-wave” pattern was unclear, it was consistent with an activation propagating from PFs to WVM (9).

Li et al (30) in their study on canines reported periods of organization during LDVF in which large wavefronts spread across almost the entire mapped region. This transition from a disorganized to an organized pattern was rapid and distinct, and during this period, activations partly arose in PF and spread to the WVM. Furthermore, mapping analysis showed that these events were consistent with repetitive endocardial focal activations, with the earliest activation site in the septal-basal electrodes (10,31). After Lugol ablation, the number of repetitive endocardial focal activations was significantly decreased, and LDVF was terminated spontaneously. Furthermore, pretreatment with the calcium chelator 1,2-bis(2-aminophenoxy)ethane-N,N,N',N'-tetraacetic acid-acetoxymethyl ester (BAPTA-AM) suppressed the genesis of repetitive endocardial focal activations and pacing-induced ventricular arrhythmia during LDVF (32).

There are a number of limitations of this study. First, the basket electrodes were widely spaced so that fine details of endocardial activation sequence were not identified. Second, intramural recordings were not made, and we were unable to determine whether the earliest activation initiated in specialized conduction system or in the working myocardiocytes of the epicardium and midwall. Third, this study only focused on the healthy canine heart, as various pathological changes affect the maintenance of LDVF. The sample size was small, so more investigations are needed to confirm our findings.

Repetitive endocardial focal activations at the LV endocardium may be associated with activation from the subendocardial PF, which may play an important role in the LDVF maintenance. The repetitive endocardial focal activations observed in this study showed a high degree of organization. Thus, different treatment strategies may be preferable during different LDVF durations.

## References

[1] Hua W, Zhang LF, Wu YF, Liu XQ, Guo DS, Zhou HL (2009). Incidence of sudden cardiac death in China: analysis of 4 regional populations.. J Am Coll Cardiol.

[2] Valenzuela TD, Roe DJ, Cretin S, Spaite DW, Larsen MP (1997). Estimating effectiveness of cardiac arrest interventions: a logistic regression survival model.. Circulation.

[3] Wik L, Hansen TB, Fylling F, Steen T, Vaagenes P, Auestad BH (2003). Delaying defibrillation to give basic cardiopulmonary resuscitation to patients with out-of-hospital ventricular fibrillation: a randomized trial.. JAMA.

[4] Tabereaux PB, Dosdall DJ, Ideker RE (2009). Mechanisms of VF maintenance: wandering wavelets, mother rotors, or foci.. Heart Rhythm.

[5] Huang J, Rogers JM, Killingsworth CR, Singh KP, Smith WM, Ideker RE (2004). Evolution of activation patterns during long-duration ventricular fibrillation in dogs.. Am J Physiol Heart Circ Physiol.

[6] Tovar OH, Jones JL (2000). Electrophysiological deterioration during long-duration ventricular fibrillation.. Circulation.

[7] Ideker RE (2007). Ventricular fibrillation: how do we put the genie back in the bottle?. Heart Rhythm.

[8] Li L, Jin Q, Huang J, Cheng KA, Ideker RE (2008). Intramural foci during long duration fibrillation in the pig ventricle.. Circ Res.

[9] Tabereaux PB, Walcott GP, Rogers JM, Kim J, Dosdall DJ, Robertson PG (2007). Activation patterns of Purkinje fibers during long-duration ventricular fibrillation in an isolated canine heart model.. Circulation.

[10] Robichaux RP, Dosdall DJ, Osorio J, Garner NW, Li L, Huang J (2010). Periods of highly synchronous, non-reentrant endocardial activation cycles occur during long-duration ventricular fibrillation.. J Cardiovasc Electrophysiol.

[11] Wu TJ, Lin SF, Hsieh YC, Ting CT, Chen PS (2006). Ventricular fibrillation during no-flow global ischemia in isolated rabbit hearts.. J Cardiovasc Electrophysiol.

[12] Cha YM, Uchida T, Wolf PL, Peters BB, Fishbein MC, Karagueuzian HS (1995). Effects of chemical subendocardial ablation on activation rate gradient during ventricular fibrillation.. Am J Physiol.

[13] Chen PS, Wolf PL, Cha YM, Peters BB, Topham SL (1993). Effects of subendocardial ablation on anodal supernormal excitation and ventricular vulnerability in open-chest dogs.. Circulation.

[14] Damiano RJ, Smith PK, Tripp HF, Asano T, Small KW, Lowe JE (1986). The effect of chemical ablation of the endocardium on ventricular fibrillation threshold.. Circulation.

[15] Durrer D, Dam RT, Freud GE, Janse MJ, Meijler FL, Arzbaecher RC (1970). Total excitation of the isolated human heart.. Circulation.

[16] Gray RA, Jalife J, Panfilov AV, Baxter WT, Cabo C, Davidenko JM (1995). Mechanisms of cardiac fibrillation.. Science.

[17] Gilmour RF, Zipes DP (1980). Different electrophysiological responses of canine endocardium and epicardium to combined hyperkalemia, hypoxia, and acidosis.. Circ Res.

[18] Friedman PL, Stewart JR, Fenoglio JJ, Wit AL (1973). Survival of subendocardial Purkinje fibers after extensive myocardial infarction in dogs.. Circ Res.

[19] Bagdonas AA, Stuckey JH, Piera J, Am NS, Hoffman BF (1961). Effects of ischemia and hypoxia on the specialized conducting system of the canine heart.. Am Heart J.

[20] Lazzara R, el-Sherif N, Scherlag BJ (1973). Electrophysiological properties of canine Purkinje cells in one-day-old myocardial infarction.. Circ Res.

[21] Robinson RB, Boyden PA, Hoffman BF, Hewett KW (1987). Electrical restitution process in dispersed canine cardiac Purkinje and ventricular cells.. Am J Physiol.

[22] Chen J, Mandapati R, Berenfeld O, Skanes AC, Jalife J (2000). High-frequency periodic sources underlie ventricular fibrillation in the isolated rabbit heart.. Circ Res.

[23] Valderrabano M, Lee MH, Ohara T, Lai AC, Fishbein MC, Lin SF (2001). Dynamics of intramural and transmural reentry during ventricular fibrillation in isolated swine ventricles.. Circ Res.

[24] Zaitsev AV, Berenfeld O, Mironov SF, Jalife J, Pertsov A (2000). M. Distribution of excitation frequencies on the epicardial and endocardial surfaces of fibrillating ventricular wall of the sheep heart.. Circ Res.

[25] Dosdall DJ, Tabereaux PB, Kim JJ, Walcott GP, Rogers JM, Killingsworth CR (2008). Chemical ablation of the Purkinje system causes early termination and activation rate slowing of long-duration ventricular fibrillation in dogs.. Am J Physiol Heart Circ Physiol.

[26] Cheng KA, Dosdall DJ, Li L, Rogers JM, Ideker RE, Huang J (2012). Evolution of activation patterns during long-duration ventricular fibrillation in pigs.. Am J Physiol Heart Circ Physiol.

[27] Huizar JF, Warren MD, Shvedko AG, Kalifa J, Moreno J, Mironov S (2007). Three distinct phases of VF during global ischemia in the isolated blood-perfused pig heart.. Am J Physiol Heart Circ Physiol.

[28] Rogers JM, Walcott GP, Gladden JD, Melnick SB, Kay MW (2007). Panoramic optical mapping reveals continuous epicardial reentry during ventricular fibrillation in the isolated swine heart.. Biophys J.

[29] Wu TJ, Lin SF, Hsieh YC, Chiu YT, Ting CT (2009). Repetitive endocardial focal discharges during ventricular fibrillation with prolonged global ischemia in isolated rabbit hearts.. Circ J.

[30] Li L, Jin Q, Dosdall DJ, Huang J, Pogwizd SM, Ideker RE (2010). Activation becomes highly organized during long-duration ventricular fibrillation in canine hearts.. Am J Physiol Heart Circ Physiol.

[31] Dosdall DJ, Osorio J, Robichaux RP, Huang J, Li L, Ideker RE (2010). Purkinje activation precedes myocardial activation following defibrillation after long-duration ventricular fibrillation.. Heart Rhythm.

[32] Wu TJ, Lin SF, Hsieh YC, Lin TC, Lin JC, Ting CT (2011). Pretreatment of BAPTA-AM suppresses the genesis of repetitive endocardial focal discharges and pacing-induced ventricular arrhythmia during global ischemia.. J Cardiovasc Electrophysiol.

